# Viral vectors in neurodegenerative diseases: immune responses and therapeutic applications

**DOI:** 10.3389/fneur.2025.1603125

**Published:** 2025-06-18

**Authors:** Yifei Wang, Siyu Mu, Fangxi Liu

**Affiliations:** ^1^Department of Neurology, The First Affiliated Hospital of China Medical University, Shenyang, China; ^2^Key Laboratory of Neurological Disease Big Data of Liaoning Province, Shenyang, China; ^3^Shenyang Clinical Medical Research Center for Difficult and Serious Diseases of the Nervous System, Shenyang, China

**Keywords:** viral vector, immune responses, neurodegenerative disease, gene therapy, AAV

## Abstract

Gene transfer-based therapies utilizing viral vectors have undergone remarkable advancements and hold significant promise in addressing neurodegenerative diseases in recent years, whose potential mechanisms include replacing or silencing pathogenic genes and delivering neurotrophic factors. Current preclinical research focuses on developing novel strategies in gene modification to combat neurodegenerative disorders. Numerous clinical trials involving viral vectors in the nervous system are either on-going or completed. Despite these advancements, progress in this field remains constrained by immune responses triggered by viral vectors, which can be triggered through innate and adaptive pathways. The present review will focus on the advances in the development and application of viral vector-based gene therapies for neurodegenerative diseases and summarize the related immune responses, aiming to provide a forward-looking perspective for this emerging arena.

## 1 Introduction

Neurodegenerative diseases (NDs) are a heterogeneous group of complex diseases characterized by neuronal loss and progressive degeneration of different areas in the nervous system (1). To date, no effective therapeutics have been developed to slow, halt, or prevent any NDs (2). As one of the therapeutic vectors widely studied, viral vectors are now routinely used as tools for studying gene function as well as developing gene-based therapies for a variety of diseases (3). Viral vectors take advantage of the ability of viruses to enter a cell, enabling direct intracellular gene product delivery (4, 5). However, increasing attention is being directed toward the immune responses induced by viral vector administration, along with the associated side effects. Although significant progress has been made over the past two decades in understanding innate and adaptive immune responses to natural infections with viral vectors or viral vector-mediated gene transfer using various types of vectors (6), further strategies are still required to mitigate the side effects caused by these immune responses.

In recent years, viral vector-mediated gene therapies have been increasingly explored as potential treatments for neurodegenerative diseases. Although preclinical and clinical data have demonstrated the relative safety and effectiveness of these *in-vivo* therapies across central nervous system (CNS) pathologies, challenges still remain in the progress of clinical translation (7). In this review, we will focus on preclinical studies using viral vector-mediated gene therapy for neurodegenerative diseases, as well as their clinical translation, and discuss the different types of immune responses elicited by viral vectors and how they translate into lack of efficacy or increased toxicity. Importantly, this review integrates advances in gene delivery with a detailed analysis of host immune reactions, highlighting the balance between therapeutic potential and immunological barriers in neurodegeneration.

## 2 Basic biology of viral vectors

Viral vectors are widely used tools for gene delivery, especially in the central nervous system (CNS). Common types include adeno-associated viruses (AAVs), retroviruses (RVs), adenoviruses (Ads), lentiviruses (LVs) (8). Viral vectors vary in their payload, cell tropism, immunogenicity and capacity and persistence of transgene induction (8). In this section, viral vectors commonly used will be discussed and key features of each vector are compared in Table 1.

**Table 1:** Key features of viral vectors.

**Vector type**	**Packaging capacity**	**Genome**	**Immunogenicity**	**Transduction efficiency**	**Gene expression stability**
Adenovirus	26–45 kb	dsDNA	High	High	Transient
Adeno-associated virus	<5 kb	ssDNA	Low	Moderate	Stable
Lentivirus	9–10 kb	ssRNA	Low	Moderate	Stable
Retrovirus	8–9 kb	ssRNA	Low	Moderate	Stable
Herpes simplex virus	50 kb	dsDNA	High	High	Transient
Stomatitis virus	6 kb	ssRNA	High	Moderate	Stable

### 2.1 Adenoviruses (Ads)

Adenoviruses have a genome size of 35–40 kb (8), which possess a capsid that accommodates a 26–45 kb linear, double-stranded DNA genome (9). Ads effectively infect dividing cells and does not integrate into the host genome (8), and they bind to various cell surface proteins to facilitate their entry into the target cells (10). The type of tissue infected largely depends on the cell tropism of the virus. It is assumed that Ads infect a broad range of cell and tissue types, including neuronal cells, dendritic cells, and hepatocytes preferentially (8). This is mainly because most human cells express the primary adenovirus receptor and the secondary integrin receptors (11, 12). Different serotypes exhibit distinct cell tropisms. For instance, Ad5 shows strong liver tropism mainly through binding of its hexon protein to coagulation factor X (10). While Ad35, a group B adenovirus, uses CD46 as its primary cellular receptor, which is broadly expressed on dendritic and hematopoietic cells, facilitating preferential infection of immune cell types (13).

Since adenoviral vectors were initially used for brain cell transduction in the early 1990s (14), they have been applied in the treatment of cancer, Parkinson's disease, and Huntington's disease (1). Ads were the first DNA virus to enter rigorous therapeutic development, largely because of its well-defined biology, genetic stability, large transgene capacity and ease of large-scale production (9). However, Ad vectors can induce high-level innate inflammatory responses within the first 24 h of transduction (15). For instance, Schaack (15) reported that adenoviral early region proteins upregulate pro-inflammatory cytokines such as IL-6 and TNF-α, even in the absence of viral replication. In spinal cord injury models, Islam and Tom observed that Ad vector delivery induced notable microglial activation and neutrophil infiltration, highlighting the potential for both therapeutic and detrimental inflammatory effects (16). Besides, they also have limitations like short gene expression of 2 weeks to several months (transient) and high risk of cytopathic effects (8, 16). Additionally, high titters can result in organ damage and even mortality (16). An intravenous administration of Ads may induce acute liver injury, as has been reported in animal models (17). Still, these disadvantages can be corrected by designing and synthesizing other novel serotypes (8). For example, the third-generation adenovirus vectors, also referred to as the helper-dependent or gutless adenovirus, can significantly reduce *in vivo* immune response compared to the first- and second-generation Ads, although high transduction efficiency and tropism are maintained (18).

### 2.2 Adeno-associated viruses (AAVs)

Since AAVs were initially discovered as a contaminant in adenovirus preparations (19), their biological characteristics and applications have been widely studied. AAVs have a single-stranded DNA, with genome DNA payloads under 5 kb (8), and have a small (~25 nm) icosahedral capsid composed of three types of structural proteins, namely, VP1, VP2, and VP3 (20). AAVs have the ability to transduce dividing and nondividing cells, but do not integrate into the host genome (8). They are capable of expressing transgenes over several months in nondividing cells (9), and provide a relatively stable expression in dividing cells as well (21). In comparison to Ads, AAVs have a long duration of gene expression *in vivo* (22). The specificity of adeno-associated virus vector-based cell and tissue targeting is determined by the capsid proteins of AAVs (9). Compared with Ads, a similar cell tropism is shown by AAVs (8). AAVs have at least 12 natural serotypes and many artificial variants, each of which exhibits different cell tropisms in the nervous system (23–26). For instance, AAV2 is one of the most extensively studied serotypes and primarily transduces neurons after direct injection into the brain parenchyma (23). AAV5 shows enhanced transduction in the striatum and cerebellum (23), while AAV8 and AAV9 are capable of crossing the blood–brain barrier (BBB) following systemic administration, thus enabling widespread CNS gene delivery (21). Notably, AAV9 preferentially targets neonatal neurons and adult astrocytes (24). AAV11 has recently been shown to enhance astrocyte-directed transduction and enable efficient retrograde labeling of projection neurons, therefore broadening its potential in circuit-specific interventions (22). These variations among AAV serotypes provide opportunities to tailor gene delivery strategies based on cell-type specificity and disease context.

AAV-mediated gene transfer has great potential as a therapeutic approach (27). Most of the currently developed AAV vectors are directed toward monogenic diseases (28), but they are also versatile and enable one to test for other CNS diseases (9). AAV vectors can lead to severe adverse effects due to off-target expression, such as hepatotoxicity (6), neurotoxicity (29), and even death in critical trials (30–32). Using AAV vectors alone does not elicit a strong immune response similar to that elicited upon using other viruses (9). AAV vectors can also induce unwanted immune responses, while about 50% of humans may have neutralizing antibodies due to previous infections (33, 34). Although certain major obstacles limit the widespread application of AAV vectors, including limited insert size, inefficient transduction, narrowed disease case, and off-target responses, they hold great potential to revolutionize the clinical management of human diseases (9). Overall, AAVs offer a relatively safe and versatile platform for CNS gene therapy, though limitations such as pre-existing neutralizing antibodies and vector immunogenicity must be addressed in clinical applications.

### 2.3 Lentiviruses (LVs)

Lentiviruses have a single-stranded RNA genome and may incorporate constructs up to 9–10 kB in size (35, 36). Lentiviral (LV) vectors can infect both dividing and non-dividing cells, such as neurons (8, 37). While it also can preserve long-term and stable transgene induction (8), which is crucial for adolescents or pediatric patients (9). Genetic engineering makes it possible to produce LVs with specific integration sites for the increased safety of use (38). Unlike Ad or AAV vectors, LV vectors rarely generate neutralizing antibodies (36). Due to their relatively low-immunogenic characteristics (35), It is reported to have low Immunogenicity and elicit no pathogenic effects (39), suggesting that this vector is relatively safe *in vivo*. LVs are capable of infecting a broad range of host cell types, allowing their use for the treatment of Parkinson's disease (PD) Alzheimer's disease (AD) (1). They have also been demonstrated as efficient gene transfer vehicles for human solid tumor cells. For example, they enabled diphtheria toxin A delivery to suppress prostate cancer xenografts (39), mediated suicide gene therapy using HSV-tk in hepatocellular carcinoma (40), and outperformed retroviral vectors in transducing ovarian cancer cells (41). Although certain problems remain to be addressed, the safe and efficient LV vectors are nonetheless considered promising as a tool for human gene therapy (9). These findings highlight the potential of LV vectors for sustained and low-immunogenic gene delivery in chronic neurodegenerative conditions.

### 2.4 Other viral vectors

Another commonly used viral vector is retroviruses (RVs), which are single-stranded RNA and integrated into the genome of the host cell (42). They can carry up to 8–9 kb of foreign DNA for transduction (43, 44). RVs can stably integrate into the target cell genome (45), so they are useful for *ex vivo* delivery of somatic cells. RVs provide a stable and efficient expression of the transgene to patients (4). They have been used in early gene therapy trials for severe combined immunodeficiency (SCID), demonstrating long-term gene correction in hematopoietic stem cells (46). However, they have limitations including immunogenic problems and inability to transduce the nondividing cells (43). Herpes simplex viruses (HSVs) are also one of the recent virus candidates in gene delivery. HSVs are an enveloped virus with a double-stranded DNA (dsDNA) Genome (4). They can usually accommodate up to 40 kb of transgenic DNA (3). Because of its neurotropic features (47), they have strong tropism for neurons. For example, HSV vectors have been studied for the delivery of neurotrophic factors in models of chronic pain and epilepsy (48). HSVs may initiate strong inflammatory response (4), and result in transient gene expression in cells infected with them (49). Vesicular Stomatitis viruses (VSVs) contain a single stranded RNA genome (50). And their cargo capacity is approximately 6 kb (50). VSVs are reported to cause humoral and cellular immune response in previous studies (50, 51). These immunological features, along with their strong cytolytic activity, support their use as oncolytic agents and viral vectors in cancer virotherapy (52). These viral vectors expand the toolkit for gene delivery in the CNS, each offering unique advantages and limitations depending on the therapeutic context.

## 3 Viral vector delivery strategies for the nervous system

There are many ways to deliver viral vectors to the brain, each varying in efficiency, target region, and biodistribution. In this section, we will introduce traditional and novel delivery strategies and their advantages and disadvantages.

### 3.1 Intraparenchymal delivery

Intraparenchymal delivery is the most used viral vector delivery method with high delivery efficiency in studies for treating neurological diseases. It's an approach that enables direct delivery to the target structure, bypassing the blood-brain barrier, and usually accomplished via a stereotactic head frame or magnetic resonance imaging guidance (53, 54). While stereotactic guided injection is frequently used for intraparenchymal delivery, risk of surgical intervention and the restricted spread of transgene expression still needs to be concerned. A clinical study on Parkinson's disease patients (NCT00400634) reported severe side effects associated with surgical procedure (55). Intraparenchymal delivery typically affects only a limited range of brain regions, with highest vector distribution and downstream gene product activity around the injection site (56, 57).

A new technology called interventional MRI-guided convection-enhanced delivery (iMRI-CED) has been introduced in recent years, which is the gold standard for verifying accurate vector delivery in real time (58). Convection-enhanced delivery (CED) has been shown to improve specificity and delivery efficiency in a safe, reliable, targeted, and homogeneous manner across the blood-brain barrier (216–219). Therefore, CED holds significant potential for broad application in the treatment of neurological diseases.

### 3.2 Intravenous delivery

Efficacy of intravenous delivery largely hinges on the ability of AAV to cross the BBB. Among the naturally existing AAVs, AAV9 is currently the most commonly used AAV serotype for CNS transduction in preclinical and clinical studies (59), which may be attributed to its active-transport mechanisms (60, 61). It has several advantages including non-invasive, widely distributed, and targeting both CNS and peripheral tissues. However, this approach also faces a few challenges. People who have been exposed to AAVs may pre-existing antibodies that can neutralize AAVs (6, 62). As it travels through the bloodstream, immune responses can diminish its efficacy (63). Besides, systemic toxicity such as off-target effects has also been found in previous studies (64, 65).

### 3.3 Intracerebralspinal fluid injection (intra-CSF)

Another method is intra-CSF injection including intra-cerebroventricular injection (ICV), intrathecal injection (IT), and intracisternal magna injection (ICM). ICV refers to direct administration into ventricles in the brain, allowing the AAV to permeate the barrier and spread widely across different brain regions (66, 67). IT delivers AAV via injection into the spinal canal through the lumbar puncture, resulting in broad coverage of the brain and spinal cord (68). ICM is a technique similar to IT, while its injection site is the cisterna magna (CM). Intra-CSF injection can bypass the BBB and spread widely within the CNS. Compared with intravenous delivery, intra-CSF injection minimizes systemic exposure and avoids neutralization by circulating antibodies. However, it has insufficient coverage, leading to limited access to deeper brain regions and the presence of surgical risks (69–71).

### 3.4 Intranasal delivery

Intranasal delivery is an alternative and non-invasive option which can directly deliver viral vectors through the nose to the brain (72). Intranasal delivery has been used in clinical studies to deliver small-molecule agents to the brain, such as nerve growth factor (NGF) and brain-derived neurotrophic factor (BDNF), thus treating neurodegenerative diseases (73–75). Intranasal delivery is easy to perform and can be repeated, even by patients themselves (72). It is also capable of bypassing the BBB (72). However, intranasal delivery holds limitations. Although it minimizes systemic exposure (76), the cells along the delivery pathways, such as olfactory sensory neurons and trigeminal nerves, may also be transduced and cause off-target effects (72).

### 3.5 FUS-BBBO

Beyond invasive approaches, focused ultrasound with microbubble-mediated BBB opening (FUS-BBBO) is an emerging non-invasive approach for viral vector delivery in treating neurological diseases. It has the potential to overcome existing delivery limitations by providing a means of non-invasive, site-specific gene transfer to the brain, offering advantages such as improved safety and targeted delivery (77–81). Various administration routes have been explored, including intravenous, intranasal, and local injection, which have expanded its applicability for gene therapy across multiple neurodegenerative disorders (81–84). Recent studies have demonstrated the successful application of FUS-BBBO in various neurodegenerative and neurological conditions. For example, AAV has been used in models of Alzheimer's disease and Huntington's disease, while HSV has been applied in glioblastoma (83–85). Importantly, FUS-BBBO may reduce systemic immune activation by enabling lower vector doses, thereby potentially lowering the risk of pre-existing neutralizing antibodies and allowing for safer repeated administration (83). Despite these advantages, FUS-BBBO has its challenges. One primary concern is still the possibility of off-target side effects and toxicity due to the intravenous administration (59). In summary, FUS-BBBO offers a promising delivery strategy that enhances brain-targeted gene therapy while potentially mitigating immune-related complications.

## 4 Immune responses against AAV injection

**Figure 1:**
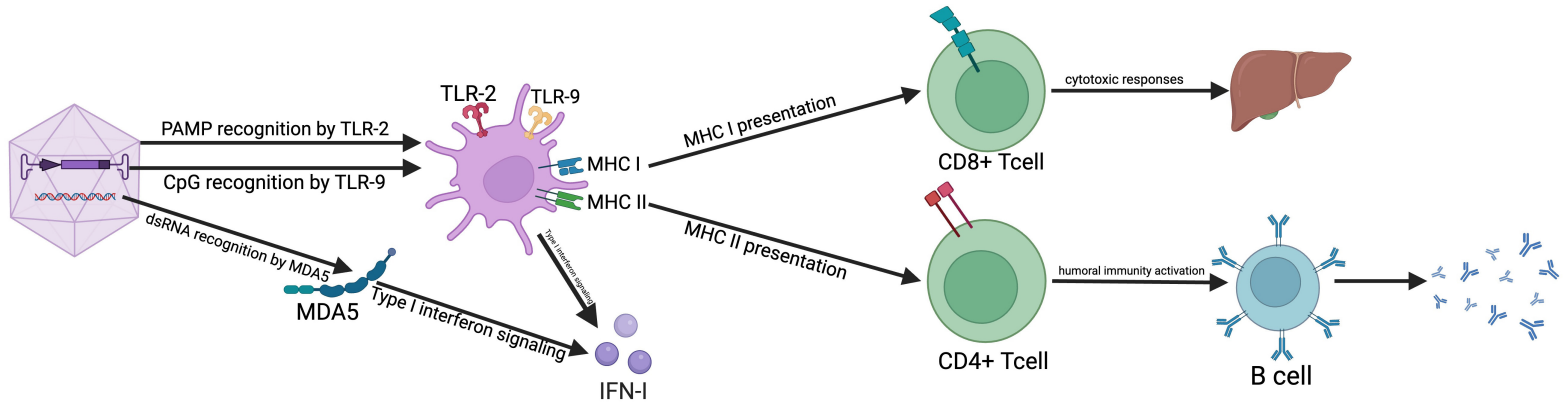
Mechanism of immune responses against AAV-mediated gene transfer. The graph shows the different type of immune responses that are elicited upon injection of AAV vectors. Toxicities are underlined and listed next to the components of the immune responses that contribute. PAMP, Pathogen-Associated Molecular Pattern; MDA5, Melanoma Differentiation-Associated protein 5; CpG, unmethylated CpG motifs; DCs, dendritic cells; dRNA, double stranded RNA; MHC, Major Histocompatibility Complex. Created in BioRender. Wang, Y. (2025) https://BioRender.com/4dt5yfl.

As the most widely studied and used viral vectors in the field of therapeutic application to neurodegenerative diseases, AAV injection was found to trigger both innate and adaptive immune responses in the current preclinical studies. As mentioned above, immune responses were also reported in other viral vectors after transduction (15, 35, 86–88). In this section, we would like to focus only on the immunogenicity of AAV vectors after administration and have further discussion. Figure 1 has summarized the main mechanisms of immune responses. Responses triggered by brain are less likely to be strong, given its immune-privileged nature (6). The responses are most likely linked to adaptive responses in most humans, except for young children who are less likely to have immune memory to AAV capsid (6).

### 4.1 Innate immune responses

Innate immunity is the first barrier against pathogens as it mounts rapidly and does not require specific adaptation to the pathogens (62). Its response depends on the recognition of pathogen-associated molecular patterns (PAMPs), which are carried by viruses or viral vectors (89). Both the capsid and the DNA components of AAV may contribute to the activation of innate immunity, resulting in adverse reactions and immune toxicity.

In the context of AAV-mediated gene transfer, one mechanism is considered to trigger inflammatory responses is a PAMP within the capsid proteins binding to Toll-like receptor 2 (TLR2) (90), positively expressed on myeloid dendritic cells. Another mechanism involves the AAV double-stranded DNA (dsDNA) genome, in particular its unmethylated CpG sequences, recognized by Toll-like receptor 9 (TLR9) (91–94). TLR9 engagement increased antigen presentation on class I major histocompatibility complex (MHC-I) and further associated with enhanced activation of AAV-specific CD8+ T cell (94–96). Further CpG Depletion of Vector Genome in animal models has shown that a reduction of the vectors' CpG motifs blunts CD8+ T cell responses without modifying B cell responses (97, 98). In addition to the vector DNA genome, double stranded RNA (dsRNA) may also participate in the induction of innate immunity from long-term AAV transduction, which could interact with melanoma differentiation-associated protein 5 (MDA5) (99). It is well known that MDA5 is cytoplasmic viral RNA sensor serving as a cytoplasmic pattern recognition receptor (PRR) and ubiquitously expressed. MDA5 is capable of activating type I interferons (type I IFNs) signaling pathways after virus infection, leading to the expression of type I IFNs (99). Besides, it is also found that AAV vectors activate type I IFNs expression in plasmacytoid dendritic cells (pDCs) of human and murine (93). TLR9 can interact with myeloid differentiation primary response gene 88 (MyD88) for downstream signaling, leading to type I IFNs production in pDCs and further promoting activation of CD8+T cells (93, 96). pDCs recognize AAV genomes via TLR9 and release type I interferons, which in turn activate conventional dendritic cells to prime capsid-specific CD8^+^ T cells—highlighting the interplay between innate sensing and adaptive immune activation (62). These studies underscore the important role of type I IFNs in bridging innate and adaptive immunity following AAV administration.

### 4.2 Adaptive immune responses

An adaptive immune response requires a longer time than innate immunity and is considered the second line of defense against pathogens. It is characterized by antigen-specific and the ability to eliminate pathogens while generating an immunological memory (62). AAV-specific adaptive responses can be induced by previous natural infection or they can be stimulated or recalled by AAV gene transfer.

After AAV vector administration, T and B lymphocytes are activated following molecular recognition of an antigen presented by antigen-presenting cells (APCs) (62). Cytotoxic CD8+T cell responding via MHC class I to AAV capsid protein can affect therapeutic efficacy (94, 95, 100), possibly by clearing AAV-transduced cells thus inducing inflammation in the target tissue (101, 102). It is also reported that increased AAV-antigen presented on capsid-derived MHC class I together with higher CD8+T cells activation in dose-dependent *in vitro* experiments (103, 104). The concurrent presentation of CD4+ T helper cells activated by capsid-derived MHC class II facilitate both humoral and cell-mediated immune responses (105). AAV-specific B cell responses induced by previous natural infections can neutralize the viral vectors before they can deliver their payload, thus precluding a successful AAV-mediated gene transfer (6). Efforts have made to avoid its neutralization by modification to the vector's capsid (106). However, it showed limited success as many of the antibodies bind to domains that are crucial for transduction (106, 107). We can see that T cells play an important part in adaptive Immune responses against viral vectors. Liver toxicity caused by T cells directed to epitopes, which bind with low affinity to MHC class I molecules, is more worthy of our attention. Therefore, cellular responses, in particular the T cells, are needed to further study and make a breakthrough.

### 4.3 Immune responses against the transgene product

Anti-transgene immune responses were documented in the clinical trials, mostly after intramuscular delivery of AAV vectors (108, 109). For example, evidence of T cell-mediated anti-transgene cytotoxic T cell responses was documented in a phase I/II trial of intramuscular gene transfer in Duchenne muscular dystrophy patients (108). The risk of antibody responses to the transgene product is influenced by many factors, including the route of vector administration, specific vector design, types of viral vectors, and vector dose. Additional host factors may include specific aspects such as tissue inflammation, target tissue and the host genetic background (105). Clinical experience indicates that immune responses directed against AAV vectors are dose dependent (110, 111). Compared to Ad and LV vectors, AAV vectors have relatively weak and transient innate response and least efficient inducer of CD8+ T cells (105). The clinical data presented suggest that disease-specific conditions, such as the ongoing inflammation in muscle dystrophies (108), are likely to increase transgene immunogenicity after gene transfer.

#### 4.3.1 Therapeutic strategies for immune responses against AAV injection

**Figure 2:**
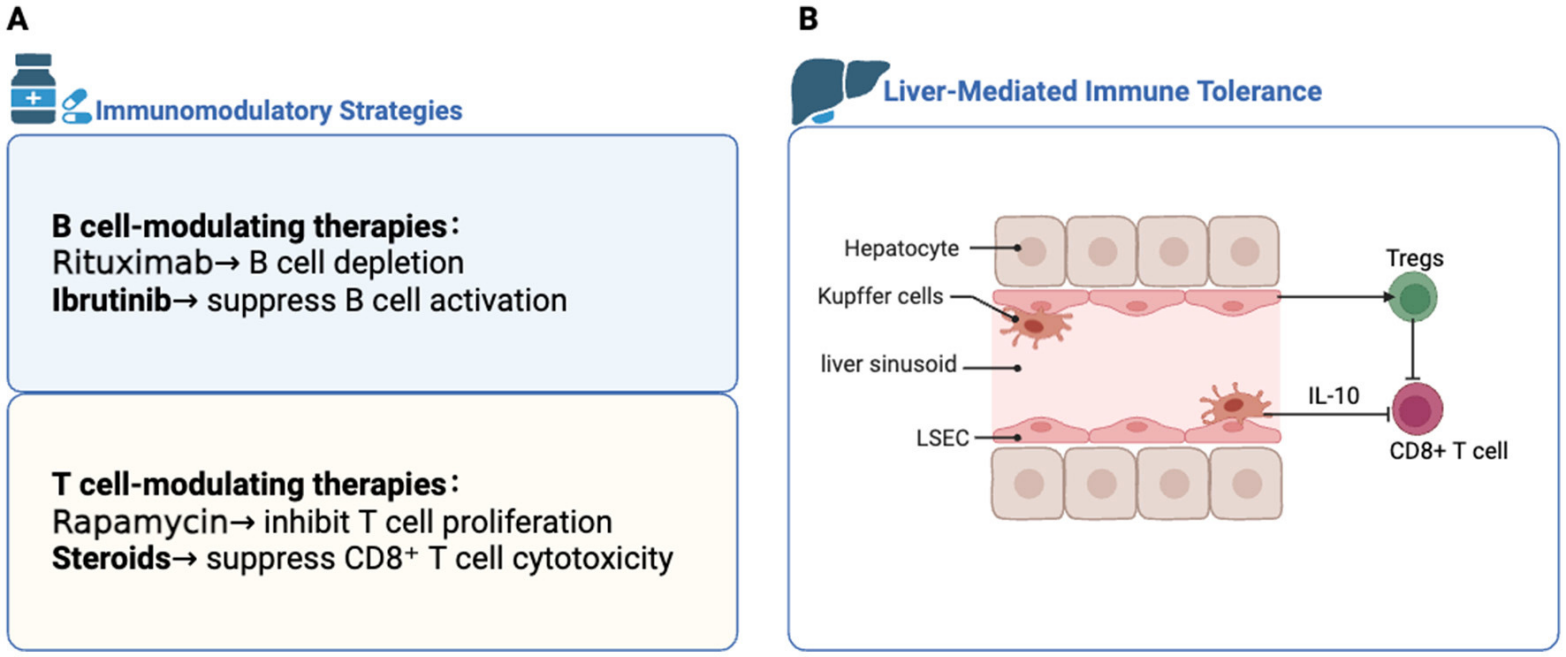
Immunomodulatory strategies and hepatic immune tolerance. **(A)** Major immunosuppressive approaches used to reduce anti-transgene immune responses, categorized by their main cellular targets. **(B)** Liver's role in immune tolerance mediated by key resident cells such as Kupffer cells and liver sinusoidal endothelial cells (LSECs). Created in BioRender. Wang, Y. (2025) https://BioRender.com/r8hrxah.

Therapies are mostly administered only once in studies nowadays, with repeated dosing precluded. According to clinical trials, AAV vectors administration leads to the development of anti-AAV IgG and NAbs (112), which could be a potential reason for preventing vector readministration. Nevertheless, preclinical studies also show that administration of immunomodulatory regimens (113) or B-cell depletion prior to gene transfer (114) can effectively block humoral immune responses against AAV vectors. These encouraging results leading to attempts in a clinical trial testing rituximab, a B-cell depleting monoclonal antibody targeting CD20 and reducing antibody responses, in combination with rapamycin (an immunosuppressant) in humans as a strategy to enable vector readministration (NCT02240407). Induction of antibodies to a transgene product could be very harmful as such antibodies could complicate traditional protein therapy. An animal study showed that, immunosuppressants such as rapamycin, which inhibits mTOR signaling to reduce T cell proliferation and inflammation. Ibrutinib inhibits Bruton's tyrosine kinase (BTK), thereby suppressing B cell activation and antibody production. Both drugs, when given alone, reduce primary antibody responses against AAV capsid (115), blunting recall responses and reducing numbers of circulating antibody-secreting plasma cells, and the combination of therapy is more effective. In a clinical trial, about a third of the patients who received Onasemnogene abeparvovec, an FDA approved AAV9 vector therapy for SMA, showed liver damage associated with an inflammatory reaction comprised mainly of CD8+ T cells, but all patients recovered after treatment with steroids, which broadly suppress immune activity and are especially effective in controlling CD8+ T cell-mediated cytotoxicity (116). However, in some cases, some patients fail to respond and still reject the AAV-transduced cells (117, 118). Besides, immunosuppression would be required for lengthy periods of time or even for the lifespan of the gene transfer recipients. Taken together, these findings emphasize the importance of different immunomodulatory strategies to improve gene therapy outcomes, including pharmacological immunosuppressants (such as rapamycin and ibrutinib) and biological agents targeting B cells (such as rituximab). Figure 2A is provided to visually summarize these therapeutic approaches.

#### 4.3.2 Hepatic immune tolerance in AAV-mediated gene therapy

Moreover, hepatic gene transfer is getting more attention in the field of immune tolerance. The liver's inherent tolerogenic nature, due to constant exposure to non-self antigens, makes it an ideal target for inducing systemic immune tolerance through gene therapy, preventing uncontrolled immune activation (119). Studies have shown that liver-directed AAV gene transfer can not only reduce systemic immune responses but also eradicate pre-existing antibodies against therapeutic proteins, such as factor VIII in hemophilia A models, highlighting the liver's role in establishing durable immune tolerance (62, 120). Therefore, liver becomes a preferred target organ for gene therapy not only for liver-specific diseases but also for disorders that require systemic delivery (121). Studies using AAV-mediated gene transfer to liver in mice and primate models indicated that hepatocyte-restricted transgene expression can induce a robust, antigen-specific peripheral tolerance (122, 123). Tolerance is induced through a combination of mechanisms. One mechanism is that Kupffer cells, a type of antigen presenting cells (APCs) resident in the liver, seem to have a less mature phenotype compared to other professional APCs (124, 125), thus leading to poor T cell-activation. Besides, Kupffer cells secret the anti-inflammatory cytokine IL-10, contributing to the suppression of CD8^+^ T cell responses (126, 127). Another important factor is liver sinusoidal endothelial cells (LSECs), who also act as professional APCs (128, 129). LSECs promote tolerance through the induction of T regulatory cells (Tregs), whose depletion in mice observed increased transgene immunogenicity (130, 131). Additional mechanisms include CD4+T cell anergy (132), T cell degradation (133) were proposed in the establishment and maintenance of liver tolerance. These key cellular mechanisms are visually summarized in Figure 2B. Overall, these findings highlight the complex interplay between innate and adaptive immunity in response to AAV vectors. Understanding how early innate signals shape long-term adaptive outcomes is essential for designing safer and more effective gene therapies. Substantially more studies are needed to address adverse immune responses.

## 5 Preclinical and clinical studies of viral vector delivery for neurodegenerative diseases treatment

In recent years, many preclinical studies and clinical trials on the application of viral vector delivery for neurodegenerative diseases have been conducted. We detail the specific pathogens and therapeutic genes involved in each disease and discuss the outcomes achieved by each way. Table 2 provides a comprehensive summary of clinical studies on neurodegenerative diseases. It is worth mentioning that, while this summary provides a broad perspective, viral vector-based gene therapy has been expanded to address diseases beyond the scope discussed here.

**Table 2:** Summary of clinical studies on neurodegenerative diseases.

**Disease**	**NCT number**	**Drug name**	**Viral vector**	**Therapeutic gene**	**Administration method**	**Phases**	**Status**	**References**
Alzheimer's Disease	NCT00876863	CERE-110	AAV2	NGF	Stereotactic injection	2	Completed	(212)
	NCT00087789	CERE-110 (AAV2-NGF)	AAV2	NGF	Stereotactic injection	1	Completed	–
	NCT05040217	AAV2-BDNF	AAV2	BDNF	Stereotactic injection	1	Recruiting	–
	NCT04133454	AAV-hTERT	AAV	TERT	Intravenous and intrathecal	1	Unknown status	–
	NCT03634007	LX1001	AAVrh10	APOE2	Intrathecal	1, 2	Recruiting	(213)
Parkinson's Disease	NCT02418598	AAV-hAADC-2	AAV2	AADC	Putaminal infusion	1, 2	Terminated	–
	NCT05603312	AAV-GAD	AAV2	GAD	Subthalamic infusion	1, 2	Recruiting	–
	NCT00400634	CERE-120	AAV2	NTN	Stereotactic surgery	2	Completed	(55)
	NCT00985517	CERE-120, AAV2-Neurturin	AAV2	NRTN	Substantia nigra and putamen surgery	1, 2	Completed	(214)
	NCT01621581	AAV2-GDNF	AAV2	GDNF	Convection-enhanced delivery	1	Completed	–
	NCT00229736	AAV-hAADC-2	AAV2	AADC	Intrastriatal infusion	1	Completed	–
	NCT00252850	CERE-120, AAV2-NTN	AAV2	NTN	Stereotactic injection	1	Completed	(215)
	NCT05822739	BBM-P002, BBM003	AAV	ND	Intracranial injection	1	Not yet recruiting	–
	NCT00643890	AAV-GAD	AAV2	GAD	Stereotactic infusion	2	Terminated	(175)
	NCT00195143	AAV-GAD	AAV2	GAD	Subthalamic infusion	1	Completed	(174)
	NCT04167540	AAV2-GDNF	AAV2	GDNF	Image-guided infusion	1	Active, not recruiting	–
	NCT01973543	VY-AADC01	AAV2	AADC	Intrastriatal injection	1	Completed	(157)
	NCT03065192	VY-AADC01	AAV2	AADC	Intrastriatal injection	1	Completed	–
	NCT03562494	VY-AADC02	AAV2	AADC	ND	2	Active, not recruiting	–
	NCT04127578	LY3884961	AAV9	GBA1	Intra-cisterna magna	1, 2	Recruiting	–
	NCT00627588	ProSavin	LV	AADC/CH1	Intraparenchymal	1, 2	Completed	(160)
	NCT01856439	ProSavin	LV	AADC/CH1	Intraparenchymal	1, 2	Terminated	(160)
Huntington Disease	NCT05541627	AB-1001	AAVrh10	CYP46A1	Intracerebral injection	1, 2	Active, not recruiting	–
	NCT05243017	AMT-130, rAAV5-miHTT	AAV5	HTT	Stereotactic infusion	1, 2	Recruiting	–
	NCT04120493	AMT-130, rAAV5-miHTT	AAV5	HTT	Stereotactic infusion	1, 2	Recruiting	–
Frontotemporal Dementia	NCT04747431	PBFT02	AAV	FTD-GRN	Intra-cisterna magna	1, 2	Recruiting	–
	NCT04408625	PR006	AAV9	FTD-GRN	Intra-cisterna magna	1, 2	Recruiting	(195)
Multiple System Atrophy	NCT04680065	AAV2-GDNF	AAV2	GDNF	ND	1	Recruiting	–

### 5.1 Alzheimer's disease (AD)

As a progressive neurodegenerative disorder, the earliest phase of Alzheimer's disease happens in parallel with accumulating amyloid β, inducing the spread of tau pathology. More than 40 Alzheimer's disease-associated genetic risk loci already have been identified, of which the APOE alleles have the strongest association with the disease (134). Preclinical and clinical studies have explored various strategies through viral vector delivery for the treatment of AD, including risky genes and related pathways.

#### 5.1.1 Targeting APOE

The common apolipoprotein E (APOE) alleles (ε4, ε3, and ε2) are important genetic risk factors for late-onset Alzheimer's disease. The primary physiological function of apoE is to mediate lipid transport in the brain and periphery (135). However, pathogenically, apoE seeds amyloid-β (Aβ) plaques in the brain with apoE4 driving earlier and more abundant amyloids (135). Studies have shown that the APOE4 allele is the strongest genetic risk factor for late-onset AD (136), increasing the risk up to 15-fold in homozygous individuals (137), whereas APOE2 is associated with a reduced risk and delayed onset of AD (138). In the brain, apoE is produced mostly by astrocytes. In one study, AAV8-GFAP-apoE, which is astrocyte-specific, was injected into apoE3-targeted replacement (apoE3-TR) or apoE4-targeted replacement (apoE4-TR) mice. In the apoE4-TR background, apoE4 decreases apoE lipidation and enhances Aβ accumulation, whereas apoE2 has the opposite effects (139). This indicates the therapeutic potential of APOE2 in the treatment of AD. In another study, AAV gene delivery of APOE2 using an AAV vector rescues the detrimental effects of APOE4 on brain amyloid pathology (140). Furthermore, Günaydin et al. have recently found that AAVrh10 delivery of novel APOE2-Christchurch (APOE2Ch) variant can better suppress amyloid and Tau pathology in “amyloid mice” and “tau mice” than APOE2 (141), which makes APOE2Ch variant a promising therapy for APOE4-associated AD. A clinical trial (NCT03634007) using AAVrh10hAPOE2 (LX1001) to treat patients with APOE4 homozygote Alzheimer's Disease was completed last year, including 15 patients. A recent interim report from the Phase 1/2 trial, showed favorable biomarker responses and good tolerability in APOE4 homozygous Alzheimer's patients, supporting continued clinical development, with further results eagerly anticipated to assess long-term efficacy.

#### 5.1.2 Expressing neurotrophic factors

Neurotrophic factors, including BDNF and NGF, have been shown to exert neuroprotective functions for AD (142). Microglia activation and reactive oxygen species (ROS) inhibition may exert neuroprotective effects against Aβ-induced neurotoxicity through NGF/TrkA signaling (143, 144). In a preclinical study, AAV2-NGF(CERE-110) was shown to be neuroprotective and neurorestorative to basal forebrain cholinergic neurons in the rat fimbria-fornix lesion and aged rat models (145). As compared to AAV2 vector, AAV2/5 vectors consistently showed higher transduction efficiency. Nagahara et al. used lentiviral gene delivery of NGF to the aged primate basal forebrain restoring cholinergic neuronal markers significantly (146). These experiments conducted in both rats and monkeys, led to the initiation of a Phase I clinical study to evaluate the safety and efficacy of NGF in Alzheimer's disease subjects. A pilot phase 1 clinical trial on 49 AD participants, AAV2-NGF was safe and well-tolerated through 24 months, but did not improve cognition (147). Further analysis of this trial revealed that it was not able to induce the cholinergic pathways which were needed for the improvement of cognition in AD patients, due to the limited spread of the injected AAV2- vectors coupled with incorrect stereotactic targeting (147). More research may be needed to find more suitable vectors to improve AAV transfection efficiency in the future.

BDNF is the most abundant neurotrophic factor in the adult brain, and its levels decline in the entorhinal cortex in AD (148). BDNF is usually combined with TrkB (149), and the BDNF/TrkB signaling promotes the accumulation of amyloid precursor protein (APP) thereby inhibiting amyloid cleavage and reducing Aβ production (150). MRI-guided infusion of AAV2-BDNF to the entorhinal cortex of the non-human primate resulted in safe and accurate targeting and distribution of BDNF to both the entorhinal cortex and the hippocampus (151). This encouraging result was further translated to a phase 1 study on early Alzheimer's Disease and mild cognitive Impairment (NCT05040217), which aims to assess the safety and distribution of AAV2-BDNF, potentially offering a disease-modifying intervention by restoring neurotrophic support to affected brain regions. This trial is currently ongoing, with surgical treatments for the Alzheimer's cohort expected to be completed by December 2027. These studies highlight the therapeutic potential of NGF and BDNF gene delivery in AD, although limitations in vector spread and targeting underscore the need for optimized delivery strategies in future clinical applications.

#### 5.1.3 Expressing TERT

Telomerase reverse transcriptase (TERT) is the enzyme responsible for maintenance of the length of telomeres (152). TERT haploinsufficiency decreases BDNF and increases amyloid-β (Aβ) precursor in murine brain (153). While increased levels of TERT in AD mouse models results in reduced Aβ accumulation, improved spine morphology, and preserved cognitive function. As an attractive target widely explored in cancer research, telomerase has gained attention for its role in neurodegeneration. A preclinical study investigating the modulation of TERT in both adult and old mice, delays physiological aging and extends longevity through AAV9-TERT gene therapy (154). Paving the way for AD patients in a phase I clinical trial currently underway, both IV and IT delivery of AAV encoding TERT are being evaluated (NCT04133454), aiming to assess safety and transgene expression in patients with mild-to-moderate AD.

### 5.2 Parkinson's disease (PD)

Parkinson's disease (PD) is one of the common neurodegenerative diseases in middle-aged and elderly people, characterized by loss of dopaminergic neurons in the substantia nigra (SN). The focal nature of SN pathology in PD has been long considered amenable to gene therapy. 3–5% of Parkinson's disease is explained by genetic causes linked to known Parkinson's disease genes, whereas 90 genetic risk variants collectively explain 16–36% of the heritable risk of polygenic Parkinson's disease (47), leading to approaches that can target genes directly linked to the disease.

#### 5.2.1 Expressing AADC

Aromatic L-amino acid decarboxylase (AADC) is an enzyme that is crucial for the synthesis of dopamine from its precursor, L-DOPA. The absence of AADC reduces dopamine synthesis in the brain. Enhanced L-DOPA to dopamine conversion leads to restored motor function and a measurable behavioral outcome in animal models of Parkinson's disease by AAV-AADC transduction (155). After infusion into parkinsonian nonhuman primate (NHP) putamen, AADC transgene expression remained unchanged at the 8-year point with no signs of neuroinflammation and other side effects (156). Transitioning from preclinical models to clinical trials, the AAV-AADC gene therapy has also shown encouraging outcomes. Magnetic resonance imaging-guided phase 1 trial of AAV2-hAADC(VY-AADC01) gene therapy for Parkinson's disease have demonstrated safety, stable expression for up to 3 years, and modest improvement in symptoms (157–159) (NCT01973543). In another clinical trial(NCT00627588 and NCT01856439), ProSavin, a lentiviral vector-based AADC gene therapy delivering all three rate-limiting enzymes (TH, AADC, and GCH1) were administered to patients with advanced Parkinson's disease, motor scores of Unified Parkinson's Disease Rating Scale (UPDRS) were significantly improved at 6-month follow-up (160).

It is worth noting that, AADC gene therapy has also been used in patients with AADC deficiency. Delivery of AAV2-hAADC to patients with AADC deficiency has been proven to be safe and effective in several clinical trials (161, 162). These encouraging results have led eladocagene exuparvovec (AAV2-AADC) to be the first FDA-approved gene therapy for the treatment of AADC deficiency. These clinical experiences in AADC deficiency have also informed dosing strategies and delivery approaches for Parkinson's disease applications. Together, these findings support the continued development of AAV2-AADC gene therapy for both Parkinson's disease and AADC deficiency.

#### 5.2.2 Expressing neurotrophic factors

Neurotrophic factors like GDNF and NRTN support neurons' growth, survival, and differentiation. GDNF, in particular, is essential for supporting dopaminergic neuron survival (163–166). Preclinical studies in both rodent and primate models have shown that AAV2-GDNF delivery to the substantia nigra and striatum increases dopaminergic neuron survival and improves motor function. In MPTP-treated rats, AAV-GDNF preserved striatal synaptic plasticity and reduced rotational asymmetry (165). In parkinsonian monkeys, AAV-GDNF enhanced dopamine activity and improved motor coordination without inducing toxicity (164, 166). Based on these findings, a Phase 1 clinical trial (NCT01621581) involving 25 patients reported that AAV2-GDNF delivery was safe and well tolerated across three escalating doses. Although the trial confirmed consistent GDNF expression, it did not provide conclusive evidence of clinical efficacy. An ongoing study (NCT04167540) is expected to further evaluate therapeutic benefit and optimal dosing. Together, these results highlight the translational potential of GDNF gene therapy for Parkinson's disease, while also emphasizing the need for further clinical validation.

Neurturin (NRTN), a member of the GDNF family of neurotrophic factors with known potential to protect and restore the function of dopaminergic substantia nigra neurons. Striatal delivery of AAV2-NRTN (CERE-120) to aged rhesus monkeys enhances activity of the dopaminergic nigrostriatal system, as indicated by increased 18F-fluorodopa uptake and tyrosine hydroxylase expression (167). CERE-120 is also proven to provide structural and functional neuroprotection and neurorestoration in MPTP-treated monkeys, leading to improvements in motor function (168). However, AAV2-neurturin delivery to the putamen and substantia nigra bilaterally in PD patients showed no significant benefit over sham surgery, possibly due to impaired retrograde transport in advanced disease stages (169). These findings highlight the discrepancy between preclinical and clinical outcomes, emphasizing the importance of timing and delivery strategies in gene therapy for PD.

#### 5.2.3 Expressing GAD

Glutamic acid decarboxylase (GAD) is an enzyme catalyzing the synthesis of GABA. GABA depletion may contribute to increased motor symptoms (170, 171), and non-motor symptoms are also related to the dysfunction of the GABAergic pathway (170).

In preclinical studies, AAV-mediated GAD expression in the subthalamic nucleus (STN) of rat and NHP models ameliorates parkinsonian behavioral phenotype (172, 173), Additionally, it demonstrated neuroprotective effects on nigral dopamine neurons, suggesting broader disease-modifying potential (172). These encouraging preclinical outcomes also lead to following phase I clinical trials (NCT00195143), in which AAV-GAD gene therapy for Parkinson's disease has proven to be safe and tolerable (174). In a phase 2 randomized trial (NCT00643890), patients in the AAV2-GAD group exhibited significantly greater improvement in motor function, as measured by UPDRS Part III scores, at both 6 and 12 months compared to the sham group (54, 175). Further study of the mechanism underlying this trail has found that only the patients who received GAD gene therapy developed a unique treatment-dependent polysynaptic brain circuit, termed as the GAD-related pattern (GADRP), which could be useful in future clinical trials for isolating true treatment responses (176). These results support the potential of GAD gene therapy not only to alleviate motor symptoms but also to offer insight into circuit-level changes associated with therapeutic efficacy.

#### 5.2.4 Targeting disease gene: GBA1

Variants in the GBA1 gene, which encodes lysosomal acid glucocerebrosidase (GCase), are among the most common genetic risk factors for Parkinson's disease and are associated with earlier onset, faster disease progression, and more severe non-motor symptoms (177). In preclinical studies, rAAV9-GBA1 was unilaterally delivered to the substantia nigra pars compacta (SNpc) in mice and NHPs. This treatment led to increased GCase activity, promoted clearance of alpha-synuclein aggregates, and enhanced survival of dopaminergic neurons, indicating potential disease-modifying effects in PD (178, 179).

Building on these findings, a Phase 1/2 clinical study (NCT04127578) using AAV9-GBA1 (LY3884961) delivered via intra-cisterna magna injection is currently ongoing to assess its safety, tolerability, and biological activity in GBA1-associated PD patients. These developments suggest that targeting GBA1 by AAV-mediated gene delivery may offer a promising therapeutic strategy for a well-defined genetic subgroup of PD, although clinical efficacy remains to be validated.

### 5.3 Huntington disease (HD)

Huntington's disease is a progressive, fatal, neurodegenerative disorder caused by an expanded CAG repeat in the huntingtin gene(HTT), which encodes an abnormally long polyglutamine (polyQ) repeat in the huntingtin protein (180). The mutant HTT protein causes selective neuronal degeneration, especially in the striatum and cortex (180). Therefore, directly targeting the HTT gene using viral vectors offers a rational and potentially disease-modifying therapeutic approach.

#### 5.3.1 Targeting HTT

As a monogenic inheritance of Huntington's disease, HTT is an appealing candidate for its gene therapy. Besides, miRNA dysregulation has been consistently reported in HD patients, transgenic HD mice, and in *in vitro* experimental models (181–184). It was also found that downregulation was dominant in the abnormal miRNA expressions (183). Consequently, delivering microRNAs that silence the mutant HTT gene has been widely studied in preclinical studies. Spronck et al. (185) performed intrastriatal injection of an AAV expressing a miRNA targeting humans HTT (AAV5-miHTT) at different doses in Q175 knock-in mice, reduced mutant HTT protein levels, and improved motor performance. Fukuoka et al. (186) introduced AAV-miR132 into the striatum of R6/2 mice, enhancing synaptic plasticity, and improving neuronal function. Keeler et al. (187) performed bilateral intrastriatal injection of AAV9-GFP-miRHtt in Q140/Q140 knock-in mice and enhanced striatal neuron survival. The animal models mentioned above are commonly used transgenetic models of Huntington's disease, and all of them are able to exhibit significant HD-like phenotypes. These strategies showed a decrease in mutant huntingtin mRNA and protein levels, lowered the mutant huntingtin aggregates in striatum and cortex, improved performance in behavioral tests, and slowed down disease progression. From the bench to the clinic, rAAV5-miHTT (AMT-130) is currently being investigated in Phase 1/2 clinical trials (NCT04120493, NCT05243017) for adults with early manifest HD. This trial involves stereotactic intrastriatal delivery and aims to evaluate safety, biodistribution, and preliminary efficacy. Together, these findings underscore the potential of AAV-mediated HTT-lowering therapy to slow disease progression in HD, especially when initiated in the early stages.

#### 5.3.2 Expressing CYP46A1

Increasing evidence suggests that CYP46A1, a gene encoding a member of the cytochrome P450 superfamily enzymes, has a role in the pathogenesis and progression of neurodegenerative disorders, and that increasing its levels in the brain is neuroprotective (188, 189). This approach is grounded in its potential to modulate cholesterol catabolism, leading to the conversion of cholesterol into 24 (S)-hydroxy-cholesterol (24S-OHC), a brain-specific oxysterol involved in cholesterol turnover. In preclinical studies, AAV-CYP46A1 infection in model mice for Huntington's disease restored cholesterol homeostasis, restored cholesterol homeostasis, enhanced cholesterol turnover, and normalized neuronal function (190, 191). Moreover, this intervention increased levels of lanosterol and desmosterol, which were found to protect striatal neurons expressing Exp-HTT from death *in vitro* (191). These findings support the hypothesis that modulating cholesterol metabolism may counteract HD pathogenesis by restoring neuronal homeostasis and enhancing cell survival. A Phase 1 trial (NCT05541627) of intracerebral bilateral injections of AAVrh10.CAG.hCYP46A1 (AB-1001) within the striatum in early manifest patients is currently ongoing.

### 5.4 Frontotemporal dementia (FTD)

GRN mutations cause frontotemporal dementia (GRN-FTD) due to deficiency in progranulin (PGRN), a lysosomal and secreted protein with unclear function, which plays roles in lysosomal homeostasis, neuronal survival, and inflammation regulation (192). Deficiency of the gene GRN results in gangliosidosis in frontotemporal dementia, causes neurodegeneration through lysosomal dysfunction, defects in autophagy, and neuroinflammation (193, 194).

Preclinical studies performed AAV-driven expression of progranulin to animal model of frontotemporal dementia due to GRN mutations (195, 196). Results have shown that it not only increased progranulin levels in the cerebrospinal fluid, but also normalized histological and biochemical markers of progranulin deficiency, including reduced lipofuscin accumulation and improved lysosomal enzyme activity (195, 196). These studies support the translation of GRN gene therapy for FTD from preclinical studies into the clinic. Interim results of a phase 1/2 trial showed preliminary insights into the safety and bioactivity of PR006 (AAV90-GRN), demonstrating increased CSF progranulin and reduced neurofilament light chain (NfL), a biomarker of neurodegeneration, while its long-term safety and potential efficacy remain to be confirmed in ongoing studies (NCT04408625, NCT04747431) (195).

### 5.5 Other neurodegenerative diseases

In other neurodegenerative diseases, GDNF can also be used in patients with Multiple System Atrophy (MSA), a rare and rapidly progressive disease involving multiple brain systems, as it has a trophic effect on the Purkinje cells (197). A Phase 1 clinical trial delivering AAV2-GDNF to the putamen in patients with either a possible or probable diagnosis of MSA has been ongoing since 2023 to evaluate its safety and potential clinical effect (NCT04680065). Amyotrophic lateral sclerosis (ALS), a progressive neurodegenerative disease affecting motor neurons, has also been a target of experimental GDNF-based therapies. Delivering a retroviral vector encoding GDNF into hind limb muscles of rodent models of ALS increased motor neuron survival and delayed the “ALS-related” decline in performance on motor tests, demonstrating functional benefits in preclinical models (198). Injection of AAV-GDNF showed evidence of retrograde transport of GDNF to motor neurons and impeded neurodegeneration in transgenic models of ALS (199). In addition to GDNF, preclinical studies using AAV-IGF1 in rodent models of ALS have also shown a significant reduction in motor neuron loss and improvement in behavior, particularly in motor function and survival extension in some models (200–202). However, further clinical translational studies are needed to assess the safety and efficacy of these approaches in humans.

In addition to adult-onset neurodegenerative disorders, recent clinical advancements in CNS-targeted gene therapy have been largely driven by rare monogenic pediatric diseases that share progressive neurodegenerative characteristics. Among them, spinal muscular atrophy (SMA) has become a flagship example of CNS-targeted gene therapy, with the FDA-approved AAV9-based Zolgensma demonstrating significant clinical efficacy. In the pivotal trial NCT02122952, systemic administration of Zolgensma, an AAV9–mediated gene therapy targeting the SMN1 gene, led to marked improvements in motor function and survival in SMA1 patients (203), findings further supported by a comparative study with a natural history cohort (204). The success of Zolgensma was further confirmed in the phase III SPR1NT trials, where presymptomatic infants with two or three copies of SMN2 achieved age-appropriate motor milestones, such as sitting and walking, underscoring the benefits of early intervention (205–207). However, the application of AAV9-based gene therapy also presents immunological challenges. In clinical trials including STR1VE and SPR1NT, immune-mediated adverse events such as transient hepatotoxicity and thrombocytopenia were commonly reported. These responses are likely due to host reactions against the AAV9 capsid or transgene product. To manage this, all patients received prophylactic corticosteroids (typically prednisolone), with liver function and hematologic parameters closely monitored and treatment adjusted accordingly (205, 207). Beyond SMA, Gene therapies for mucopolysaccharidoses (MPS, e.g., NCT03580083) and Rett syndrome (e.g., NCT06856759) are actively being explored. While most treatments remain in early or mid-stage trials with limited clinical efficacy so far, these efforts provide important proofs of concept for CNS-targeted gene therapy. Their progress offers valuable lessons on delivery strategies and immune response management, paving the way for future applications in adult neurodegenerative diseases.

## 6 Discussion

Immune responses-mediated toxicity caused by viral vectors affects the efficacy of gene therapy. Although immunosuppression has been used successfully to blunt some of the viral vector-induced immune responses, it has failed in other cases. Thus, the design of more effective and less immunogenic viral vectors is needed, for example by incorporating DNA-depleted AAV capsids (empty capsids) (208, 209). In addition, further optimized viral vectors could aim to improve the efficiency of transduction and the stability and persistence of gene expression, thus significantly expanding the application scope of viral vectors. With the rapid development of gene therapy, gene editing technologies have played a crucial role. Viral vectors are able to precisely deliver gene editing tools, such as CRISPR-Cas9, to target cells, particularly in *in vivo* experiments (210, 211). In this way, researchers can precisely manipulate specific genes, such as knocking out harmful genes, inserting functional ones or repairing mutations. This not only broadens the potential applications of gene editing technology but also plays an important role in basic research and disease treatment. Traditional strategies to deliver viral vectors lack spatial precision and thus cannot target regionally defined neural circuits. New and non-invasive methods, like FUS-BBBO, are increasingly being adopted and more novel delivery methods are to be explored.

As the safety and efficacy of virus as a vector are established, future studies examining vector-mediated delivery of different molecules for treating different neurodegenerative diseases is to be proved. The promising results from preclinical studies and the encouraging data from ongoing clinical trials provide a hopeful outlook for the application of gene therapy in neurological diseases. Still, significant challenges need to be resolved before gene therapy for neurodegenerative diseases becomes widely accepted. In some diseases, although some diseases have reproducibly positive data in preclinical models, clinical trials didn't show significant results. It remains to be determined if lack of success in clinical trials reflects a lack of biological effect of intervention, limitations of the technical delivery methods or failure to reverse the course of the advanced disease at the time of intervention. For neurodegenerative diseases, the disease may have progressed to a point with limited possibilities of intervention, that's why early diagnosis is crucial for attaining a better outcome. Since available clinical trials have limited follow-up data, future research should focus on long-term studies to better understand the efficacy and safety of these therapies.

## 7 Conclusion

In summary, we can see immune responses after vector administration and immune system-mediated toxicity continue to challenge the success of gene transfer by viral vectors especially when high doses are required to correct the targeted genetic disease. Some novel approaches, such as hepatic gene transfer, can mitigate immune toxicity to a certain extent. Given that numerous mechanisms and molecules have been extensively studied as potential protective interventions for neurodegenerative diseases, viral vector-mediated gene therapies, particularly those utilizing AAV vectors, have emerged as a highly promising approach in both preclinical and clinical studies. Before gene therapy for neurodegenerative diseases can be widely adopted, significant hurdles must be overcome by improving evidence-based strategies, mitigating immune-related adverse effects, and conducting larger clinical trials with longer follow-up periods.
